# Multiple intestinal lymphangiomas with episodic hemorrhage requiring partial laparoscopic resection: a case report

**DOI:** 10.1186/s40792-022-01411-y

**Published:** 2022-03-31

**Authors:** Hiroka Kondo, Takeshi Ohki, Shimpei Ogawa, Teppei Omori, Hiromi Onizuka, Yoji Nagashima, Shigeki Yamaguchi

**Affiliations:** 1 Department of Surgery, Institute of Gastroenterology, Tokyo Women’s Medical University, 8-1, Kawada-cho, Shinjuku-ku, Tokyo, 162-8666 Japan; 2 Department of Gastroenterology, Tokyo Women’s Medical University, 8-1, Kawada-cho, Shinjuku-ku, Tokyo, Japan; 3 Department of Surgical Pathology, Tokyo Women’s Medical University, 8-1, Kawada-cho, Shinjuku-ku, Tokyo, Japan

**Keywords:** Lymphangioma, Mesenteric, Intestinal, Anemia, Laparoscopic-assisted surgery

## Abstract

**Background:**

Lymphangioma is a non-epithelial tumor marked by aggregates of abnormally dilated lymphatics. Mesenteric occurrences account for < 1% of all cases, and < 0.05% involve the gastrointestinal tract. Most are confined to children, rarely affecting adults.

**Case presentation:**

Herein, we describe an elderly Japanese woman with anemia, hypoalbuminemia, and episodic bleeding due to multiple intestinal lymphangiomas. Abdominal computed tomography revealed multiple low-density defects of mesentery, with areas of intermediate (T1 images) or high (T2 images) signal intensity similarly dispersed in magnetic resonance scanning sequences. Single-balloon enteroscopy was undertaken, enabling identification and tattooing of a small intestinal bleeding source. Laparoscopy-assisted resection at this site served to control related hemorrhage, removing a histologically confirmed hemolymphangioma. Having recovered uneventfully, the patient remained stable 2 months postoperatively.

**Conclusions:**

Although rare in adults, mesenteric or gastrointestinal lymphangiomas must be considered in a setting of anemia and hypoalbuminemia. Complete resection is advantageous to improve patient symptoms, but limited resection of multiple lesions may be equally effective.

## Background

Lymphangioma is a non-epithelial growth composed of dilated lymphatic channels [1]. Mesenteric lesions are quite rare (< 1% of such tumors) [2] and may remain asymptomatic, present as distinct abdominal masses, or culminate in acute abdomen [2, 3]. Once bleeding occurs, hemostasis is largely achieved through upper gastrointestinal (GI) endoscopy or enteroscopy, reserving surgery for difficult cases [4–8]. Herein, we report an elderly woman with multiple mesenteric and intestinal lymphangiomas that bled repeatedly. The culprit site was tattooed during small bowel enteroscopy, and laparoscopic-assisted surgical resection followed.

## Case presentation

A 77-year-old Japanese woman with chronic anemia (20 + years) had engaged elsewhere in supplementation for presumed iron deficiency. Her anemic condition seemed to abate until 6 years prior, at which point another episode prompted upper GI endoscopy and bone marrow examination. No abnormalities were apparent at the time, but fecal testing was positive for occult blood. Eventually, she was referred to our hospital in consultation. Subsequent colonoscopy and enteroscopy revealed numerous mesenteric and intestinal lymphangiomas in conjunction with bleeding. Given her fluctuating status during medical treatment, often reliant on transfusions, surgical control of blood loss was the therapeutic goal.

Upon admission, the patient (height, 145 cm; weight, 50.4 kg) had a body mass index (BMI) of 24 kg/m^2^. Conjunctival pallor and lower leg edema were evident, but her abdomen was flat, soft, and non-tender. Other than very low hemoglobin (6.7 g/dL) and albumin (2.5 g/dL) levels, initial laboratory diagnostics were unremarkable. Her overall condition had been sustained through supportive measures, administering blood and albumin.

**Fig. 1:**
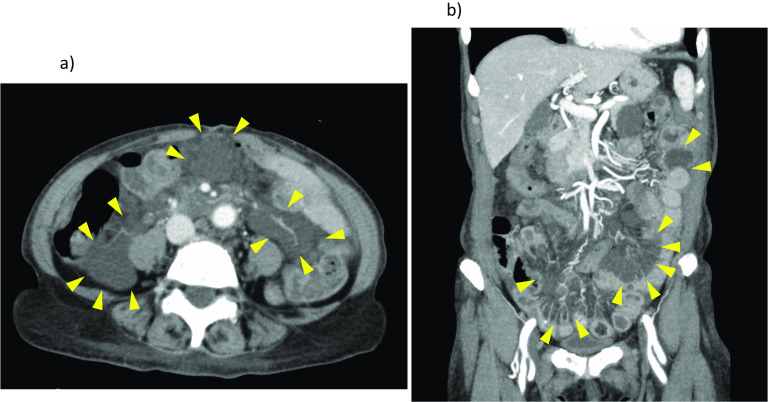
Enhanced abdominal computed tomography: Multiple low-density areas of mesentery in **a** axial and **b** coronal views, devoid of contrast effect (arrowhead)

**Fig. 2:**
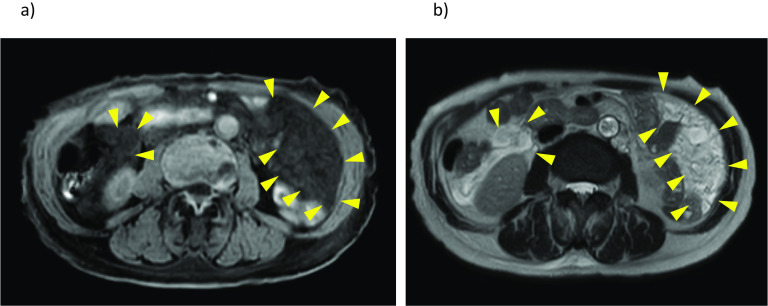
Abdominal magnetic resonance imaging study: Multiple lesions of **a** T1-weighted and **b** T2-weighted sequences, showing respective signal intensities (T1, intermediate; T2, high) (arrowhead)

**Fig. 3:**
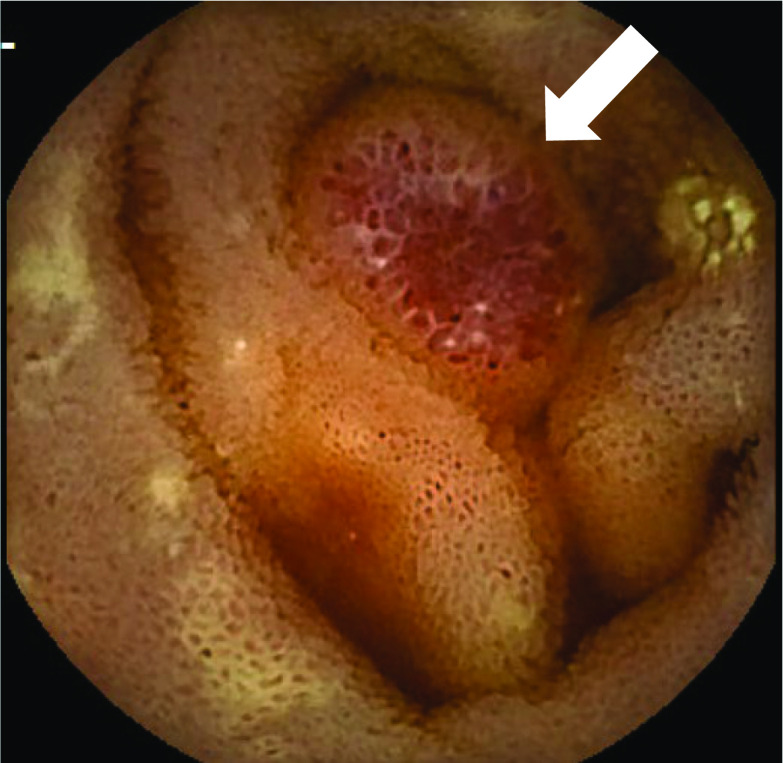
Capsule enteroscopic delineation of multiple lymphangiomas at jejunum and terminal ileum: the surface was yellowish-white with reddish color and finely granular (arrow)

**Fig. 4:**
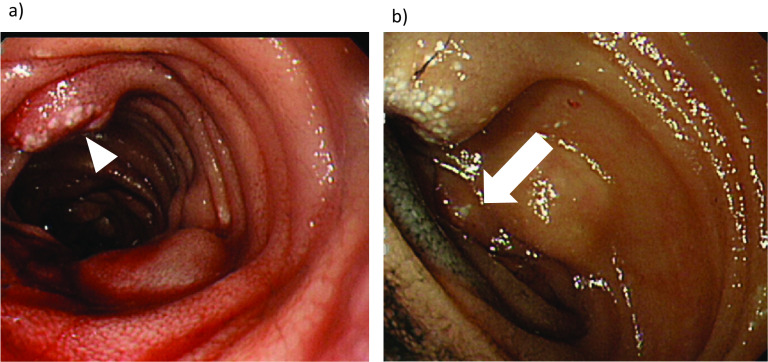
**a** Enteroscopy revealed an area that appeared to be bleeding heavily (arrowhead). **b** Enteroscopic tattoo applied to the same area (arrow)

Abdominal computed tomography (CT) revealed multiple low-density mesenteric defects, devoid of contrast effect (Fig. 1). Findings of magnetic resonance imaging (MRI) are shown in Fig. 2. There were many areas of intermediate (T1) or high (T2) signal intensity in respective scanning sequences. Capsule enteroscopy confirmed a multiplicity of lymphangiomas along jejunum and near terminal ileum (Fig. 3). Single-balloon enteroscopic tattooing was done, marking a suspected jejunal bleeding source for later operative intervention (Fig. 4).

**Fig. 5:**
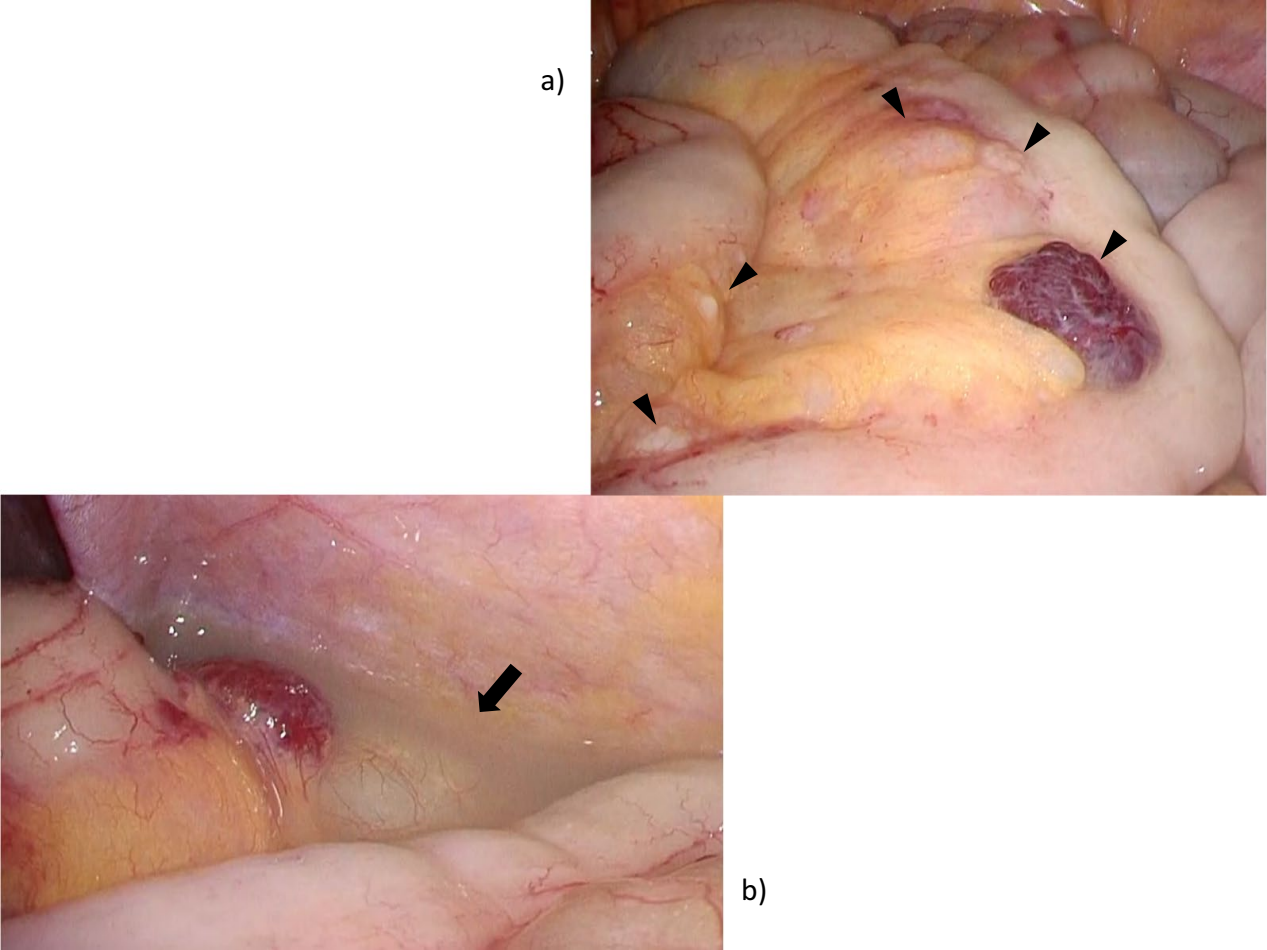
Operative findings: **a** array of yellowish, cystic lesions and hemangiomatous tumors dispersed along small intestine and mesentery (arrowhead) and **b** small volume of grayish-white, slightly cloudy ascites (arrow)

Hemostasis was ultimately achieved through laparoscopic-assisted surgery. A 4-cm umbilical incision was made, installing a multi-flap gate for converter attachment and 12-mm port insertion. CO_2_ insufflation (10 mmHg) produced a pneumoperitoneum sufficient for viewing the abdominal cavity. Many yellowish, cystic growths and hemangiomatous nodules studded the small intestine and mesentery, accompanied by low-volume, grayish-white, and slightly cloudy ascites (Fig. 5). Extracorporeal operation proceeded, once the above concluded. Having inspected and palpated the entire small intestine up to ligament of Treitz, it appeared that the preponderance of cystic mesenteric and intestinal lesions were jejunal. Their sheer number prohibited complete resection, but partial excision was still feasible. We thus removed intestine and adjacent mesentery at the point of tattoo (to control bleeding), performing functional end-to-end anastomosis. Operative time was 114 min, blood loss was 3 ml, and no blood was transfused during or after the operation.

**Fig. 6:**
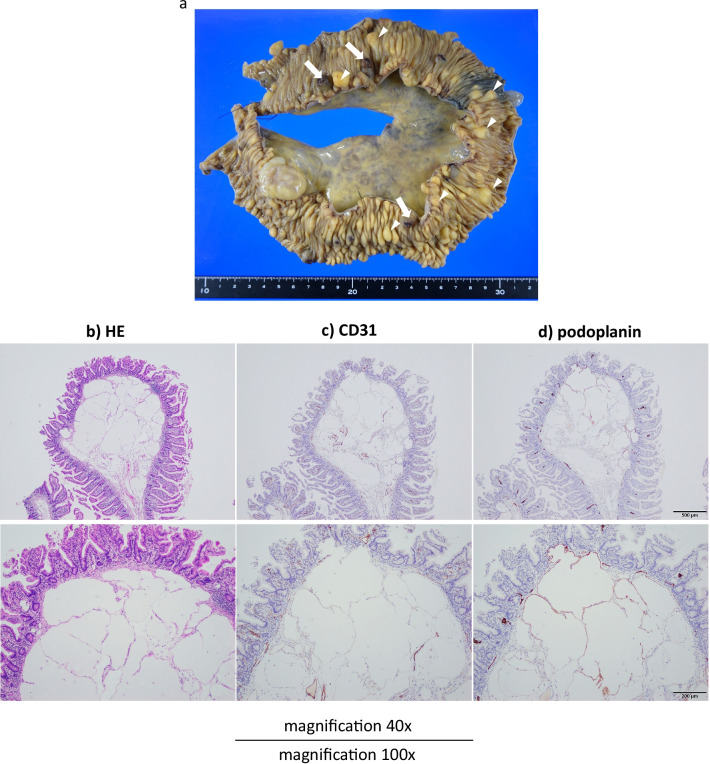
Gross and microscopic tumor presentation: **a** multiple yellowish-white submucosal lesions (arrowhead on typical ones), and some with traces of post-hemorrhage (arrow); **b** hematoxylin and eosin stain, magnification ×40 and ×100; **c** CD31 immunostain, magnification ×40 and ×100; and **d** podoplanin immunostain, magnification ×40 and ×100. These lymphoid structures were proven to be positive for podoplanin by immunostaining, and negative for CD31

The anemia stabilized postoperatively. In Fig. 6, multiple yellowish-white submucosal lesions are grossly visible, seen as a profusion of dilated lymphatic vessels in hematoxylin and eosin-stained histologic sections. These lymphoid structures were proven to be positive for podoplanin by immunostaining, and negative for CD31. The patient recuperated without incident and was discharged 8 days after surgery. At postoperative Month 2, no worsening anemia or hyperalbuminemia had developed.

## Conclusions and discussion

Mesenteric lymphangiomas are rare congenital malformations of the lymphatic system, presenting as non-epithelial, neoplastic proliferations of dilated lymphatic channels [1]. The most common sites of origin are head and neck region or axilla, together contributing > 95% of cases [9]. GI lesions account for < 0.05% [10]. Definitive diagnosis relies on histologic features, using depth and size of abnormal lymphatics to recognize four tumor subtypes as follows: capillary, cavernous, cystic (hygroma), or hemolymphangioma (mixed vascular/lymphatic elements) [11]. Lesions in this patient corresponded with the latter. According to past reports, lymphangiomas resected for hemostasis have been conspicuous growths [4, 6, 12–14], harboring putative vascular constituents that promote blood loss.

Most mesenteric lymphangiomas (65%) are discovered at birth, and nearly all (90%) are reportedly diagnosed by age 2 [15]. Lymphangiomas of small intestine rarely occur, comprising 1–2% of benign small bowel tumors [16]. Without benefit of enteroscopy, they defy preoperative detection [17]. This case is fairly emblematic, failing to amass conclusive evidence despite longstanding anemia and repeated diagnostic testing. Enteroscopy is thus a worthwhile pursuit in any patient with obscure yet progressive anemia and hypoalbuminemia, bearing lymphangioma in mind.

While generally benign in nature, such tumors may pervade mesenteric roots and peripheries (at intestinal wall) or invade surrounding organs, with potentially life-threatening complications. Traumatic rupture (causing intra-abdominal or intracavitary bleeding and anemia), ischemic tissue necrosis, intestinal gangrene (due to axial torsion), and intermittent intestinal obstruction are possible [18, 19]. GI bleeding was episodic in our patient and difficult to control by medical means. The alternative was surgical resection. However, other published reports [4, 6, 8, 20–22] have warned of the elusiveness posed by bleeding sites amidst multiple lesions.

Ugwonali et al. have used intraoperative enteroscopy to identify and excise sources of hemorrhage in this setting, achieving good results [23]. Morris-Stiff et al. have also installed preliminary tattoos during small bowel endoscopy as flags for resection [7]. Our patient had numerous growths, often adjacent to intestinal wall, limiting the traceability of active bleeding by intraoperative palpation alone. Enteroscopy performed in advance of surgery to pinpoint and tattoo a suspected bleeding site helped expedite intraoperative resection of the culprit segment.

Complete resection of a localized lymphangioma is of course ideal [19, 24]. However, partial resection constitutes a valid approach to multiple lesions, offering substantial volume reduction and serving to improve patient quality of life [25]. Residual neoplastic involvement is a lingering concern that must be addressed through careful follow-up monitoring.

Herein, we describe a patient with multiple mesenteric and intestinal lymphangiomas who experienced chronic episodic bleeding. The situation was resolved through enteroscopic tattoo and laparoscopic-assisted surgical resection. In adults, mesenteric and GI lymphangiomas are rare occurrences to bear in mind as underlying causes of anemia and hypoalbuminemia. Although complete resection is best for symptom improvement, partial resection of identified bleeding points may likewise enhance patient quality of life. Preliminary enteroscopic tattooing is a means of facilitating minimally invasive surgery in these circumstances.

## Data Availability

All data generated or analyzed during this investigation are included in the published manuscript.

## Abbreviations

BMI: Body mass index
CT: Computed tomography
GI: Gastrointestinal
MRI: Magnetic resonance imaging

## References

[1] Chen J, Du L, Wang DR (2018). Experience in the diagnosis and treatment of mesenteric lymphangioma in adults: A case report and review of literature. World J Gastrointest Oncol.

[2] Mendez-Gallart R, Solar-Boga A, Gomez-Tellado M, Somoza-Argibay I (2009). Giant mesenteric cystic lymphangioma in an infant presenting with acute bowel obstruction. Can J Surg.

[3] Guth S, Gocke C, Gebhardt J, Schwenk W, Caselitz J, Bamberger CM (2010). Mesenterial lymphangiolipoma - a rare finding in an asymptomatic patient. Med Klin (in German).

[4] Antonino A, Gragnano E, Sangiuliano N, Rosato A, Maglio M, De Palma M (2014). A very rare case of duodenal hemolymphangioma presenting with iron deficiency anemia. Int J Surg Case Rep.

[5] Bucciero F, Marsico M, Galli A, Tarocchi M (2015). Small bowel lymphangioma: A rare case of intestinal bleeding. Dig Liver Dis.

[6] Fang YF, Qiu LF, Du Y, Jiang ZN, Gao M (2012). Small intestinal hemolymphangioma with bleeding: a case report. World J Gastroenterol.

[7] Morris-Stiff G, Falk GA, El-Hayek K, Vargo J, Bronner M, Vogt DP (2011). Jejunal cavernous lymphangioma. BMJ Case Rep.

[8] Lim DR, Kuk JC, Kim T, Shin EJ (2018). Surgery of multiple lymphangioma in small bowel: a rare case report of chronic gastrointestinal bleeding. Ann Surg Treat Res.

[9] Fukuda S, Hossain Z (1977). Cystic lymphangioma of the mediastinum and the small bowel: a case report and a review of literature. Del Med J.

[10] Peters FT, Rutgers EJ, Driessen WM (1987). Intra-abdominal lymphangiomatous cysts in adults. Neth J Med.

[11] Kosmidis I, Vlachou M, Koutroufinis A, Filiopoulos K (2010). Hemolymphangioma of the lower extremities in children: two case reports. J Orthop Surg Res.

[12] Blanco Velasco G, Tun Abraham A, Hernandez Mondragon O, Blancas Valencia JM (2017). Hemolymphangioma as a cause of overt obscure gastrointestinal bleeding: a case report. Rev Esp Enferm Dig.

[13] Gomez-Galan S, Mosquera-Paz MS, Ceballos J, Cifuentes-Grillo PA, Gutierrez-Soriano L (2016). Duodenal hemangiolymphangioma presenting as chronic anemia: a case report. BMC Res Notes.

[14] Ralston M (1961). Lymphangioma-haemangioma of the jejunum: a rare cause of alimentary tract bleeding. Aust N Z J Surg.

[15] Geraci G, Sciume C, Pisello F, Volsi FL, Facella T, Tinaglia D, Arnone E, Modica G (2006). Mesenteric cyst lymphangioma; a case report and literature review. Ann Ital Chir.

[16] River L, Silverstein J, Tope JW (1956). Benign neoplasms of the small intestine; a critical comprehensive review with reports of 20 new cases. Surg Gynecol Obstet.

[17] Yamamoto H, Sekine Y, Sato Y, Higashizawa T, Miyata T, Iino S, Ido K, Sugano K (2001). Total enteroscopy with a nonsurgical steerable double-balloon method. Gastrointest Endosc.

[18] Kim SH, Kim HY, Lee C, Min HS, Jung SE (2016). Clinical features of mesenteric lymphatic malformation in children. J Pediatr Surg.

[19] Losanoff JE, Kjossev KT (2005). Mesenteric cystic lymphangioma: unusual cause of intra-abdominal catastrophe in an adult. Int J Clin Pract.

[20] Griffa B, Basilico V, Feltri M, Griffa A (2006). Submucosal jejunal lymphangioma: an unusual case with obscure gastrointestinal bleeding in an adult, detected by video-capsule endoscopy and treated by laparoscopy. Minerva Chir.

[21] Tan B, Zhang SY, Wang YN, Li Y, Shi XH, Qian JM (2020). Jejunal cavernous lymphangioma manifested as gastrointestinal bleeding with hypogammaglobulinemia in adult: A case report and literature review. World J Clin Cases.

[22] Yang J, Zhang Y, Kou G, Li Y (2020). Jejunum Hemolymphangioma Causing Refractory Anemia in a Young Woman. Am J Gastroenterol.

[23] Ugwonali O, Coady M, Saxena R, Robert M, Horowitz N, Topazian M (2000). Intraoperative enteroscopy for diagnosis of a bleeding jejunal lymphangioma. J Clin Gastroenterol.

[24] Weeda VB, Booij KA, Aronson DC (2008). Mesenteric cystic lymphangioma: a congenital and an acquired anomaly? Two cases and a review of the literature. J Pediatr Surg.

[25] Kumar B, Bhatnagar A, Upadhyaya VD, Gangopadhyay AN (2017). Small Intestinal Lymphangioma Presenting as an Acute Abdomen with Relevant Review of Literature. J Clin Diagn Res.

